# Measuring the effectiveness of in-hospital and on-base Prevent Alcohol and Risk-related Trauma in Youth (P.A.R.T.Y.) programs on reducing alcohol related harms in naval trainees: P.A.R.T.Y. Defence study protocol

**DOI:** 10.1186/s12889-017-4330-8

**Published:** 2017-05-02

**Authors:** Jason Watterson, Belinda Gabbe, Paul Dietze, Jennifer Thompson, Michael Oborn, Jeffrey V. Rosenfeld

**Affiliations:** 1 Department of Epidemiology and Preventive Medicine, School of Public Health and Preventive Medicine, Monash University, Melbourne, Australia; 2 Department of Neurosurgery, Alfred Hospital, Melbourne, Australia; 3 National Trauma Research Institute, Alfred Health, 553 St Kilda Road, Melbourne, VIC 3004 Australia; 4 Royal Australian Navy, Canberra, Australia; 5 Royal Australian Navy Reserve, Canberra, Australia; 6 Australian Army, Canberra, Australia; 7 Centre for Population Health, Burnet Institute, Melbourne, Australia

**Keywords:** Military personnel, Awareness, Risk-taking, Alcohol

## Abstract

**Background:**

Reducing alcohol related harms in Australian Defence Force (ADF) trainees has been identified as a priority, but there are few evidence-based prevention programs available for the military setting. The study aims to test whether the P.A.R.T.Y. program delivered in-hospital or on-base, can reduce harmful alcohol consumption among ADF trainees.

**Methods/design:**

The study is a 3-arm randomized controlled trial, involving 953 Royal Australian Navy trainees from a single base. Trainees, aged 18 to 30 years, will be randomly assigned to the study arms: i. in-hospital P.A.R.T.Y.; ii. On-base P.A.R.T.Y.; and iii. Control group. All groups will receive the routine ADF annual alcohol awareness training. The primary outcome is the proportion of participants reporting an Alcohol Use Disorders Identification Test (AUDIT) score of 8 or above at 12 months’ post-intervention. The secondary outcome is the number of alcohol related incidents reported to the Royal Australian Navy (RAN) in the 12 months’ post-intervention.

**Discussion:**

This is the first trial of the use of the P.A.R.T.Y. program in the military. If the proposed intervention proves efficacious, it may be a useful program in the early education of RAN trainees.

**Trial registration:**

Australian New Zealand Clinical Trials Registry (ANZCTR): ACTRN12614001332617, date of registration: 18/12/2014 ‘retrospectively registered’.

**Electronic supplementary material:**

The online version of this article (doi:10.1186/s12889-017-4330-8) contains supplementary material, which is available to authorized users.

## Background

Almost all societies that consume alcohol experience related health and social problems [1]. Excessive alcohol consumption, and associated harm, is a major contributor to morbidity and mortality in Australia and other developed countries [1–3]. Therefore, interventions to reduce excessive consumption and associated harm are needed.

To reduce the occurrence and cost of substance abuse problems, it is important to intervene early before harmful patterns of substance use are established [4, 5]. One intervention that is commonly used in an effort to reduce alcohol related harm in young people is the P.A.R.T.Y. program. P.A.R.T.Y. is an in-hospital, injury awareness and prevention program which originated in Canada in 1986, and now operates in over 100 sites around the world.

P.A.R.T.Y. is a full day program that involves interaction, generally of school groups, with emergency services personnel, health professionals, and patients who have experienced trauma and survived. Holding the program within a hospital environment is expected to leave a significant and lasting impression of the consequences of preventable trauma and risk taking behaviours [6, 7] (Additional file 1: Appendixes 1 and 2).

Case-control studies have shown that attendance at P.A.R.T.Y. reduced major trauma presentations in senior school students in Canada [6], and recidivism in juvenile justice young offenders [7]. Further, a pilot cohort study explored the feasibility of delivering the P.A.R.T.Y. program to 108 ‘at risk’ naval trainees, and found that 12 months after program participation, 14% of participants had been reported for an alcohol related incident, with the rate of reported alcohol related incidents following the program higher for participants who had a pre-program incident on record (Watterson, Gabbe, Oborn, Thompson and Rosenfeld: Piloting an injury awareness and education program for reducing alcohol related harm in Navy Trainees, Submitted).

While these observational studies provide promising supporting evidence for P.A.R.T.Y., the efficacy of this program in reducing risk taking behaviour and alcohol related harms has not been established. Our study will test whether participation in either an in-hospital or on-base version of the P.A.R.T.Y. program leads to a reduction in prevalence of risky drinking (hazardous/harmful, measured using the Alcohol Use Disorders Identification Test, AUDIT) [8–10] at 12 months’ post-intervention in a randomised controlled trial (RCT). We expect that naval trainees who participate in an on-base, or in-hospital, P.A.R.T.Y program will have a lower prevalence of hazardous or harmful drinking behaviour, compared to naval trainees who do not attend the P.A.R.T.Y. program.

## Methods

### Setting

Participants for this study will be recruited from Initial Entry Trainees who are completing their specialist training at the RAN’s key training establishment in Victoria. The intervention (P.A.R.T.Y.) will be delivered at two settings; at The Alfred (‘in-hospital’) and at the participating naval base (‘on-base’).

### Ethics approval and trial registration

Ethical approval for the study was obtained from the Australian Defence (739–13), Alfred Health (155/16) and Monash University (CF14/1667–2,014,000,783) Human Research Ethics Committees. The trial is registered in the Australian New Zealand Clinical Trials Registry (ANZCTR) ACTRN12614001332617.

### Study design

**Fig. 1:**
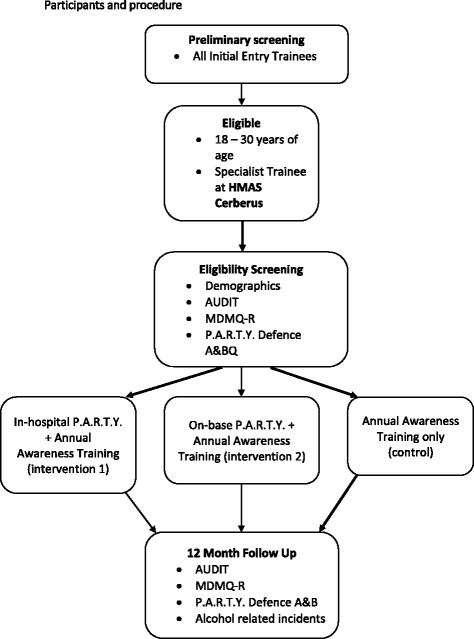
Participant flow chart (AUDIT, Alcohol Use Disorders Identification Test; MDMQ-R, Modified Drinking Motives Questionnaire – Revised; P.A.R.T.Y. Defence A&BQ, Prevent Alcohol and Risk-related Trauma in Youth Defence Attitudes and Behaviours Questionnaires)

In this non-blinded RCT, participants will be randomised to one of three arms:

i. In-hospital P.A.R.T.Y. program and annual alcohol and other drugs awareness training;
ii. On-Base P.A.R.T.Y. program and annual alcohol and other drugs awareness training; and
iii. Annual alcohol and other drugs awareness training only (Fig. 1).

### Participants and procedure

#### Inclusion and exclusion criteria

Naval trainees will be eligible for inclusion in the study if they meet the following criteria:

i. Stationed at the participating naval base for training of at least 12 weeks’ duration following initial entry training (IET), and;
ii. No alcohol related incident on their service record since joining the RAN.

### Procedures

#### Screening

Participants will be recruited from all initial entry trainees based at the participating naval base. The recruitment process is scheduled from March 2014 to May 2016 and will be conducted by the research project staff. Eligible participants will be provided with a baseline screening pack which includes a participant information and consent form, a copy of the Australian Defence Human Research Ethics Committee – Guidelines for Volunteers information sheet, a demographics form, and three screening questionnaires:

i. Alcohol Use Disorders Identification Test (AUDIT) is a 10-item questionnaire validated in a cross-national study initiated by the World Health Organization (WHO) in 1982 [10]. AUDIT is used to screen for hazardous and harmful drinking, with scores ≥8 typically taken to indicate hazardous or harmful drinking [10–12].
ii. Modified Drinking Motives Questionnaire – Revised (MDMQ-R), first conceptualised in 1988 [13] to describe the motivators for the use of alcohol. The MDMQ-R consists of 28 items, each of these contribute to one of five subscales: social, coping-anxiety, coping-depression, enhancement and conformity; and
iii. P.A.R.T.Y. Defence Attitudes and Behaviours Questionnaire, developed specifically for this study comprising questions stemming from both the annual alcohol poll conducted by the Foundation for Alcohol Research and Education (FARE) and recommendations from the review of the use of alcohol in the Australian Defence Force (ADF) Additional file 1: Appendix 1 [14, 15].

#### Randomisation

Following completion of consent forms and baseline screening measures, consented participants will be randomly allocated in a 1:1:1 ratio to one of the three trial arms. Randomisation will be stratified by gender in block sizes of 3, 6, 9, 12 or 15 patients. Randomisation will take place at an individual level. The allocation will be performed by a designated person not involved in the conduct of the programs, measurement of the outcomes, or analysis of the data. An opaque envelope containing computer-generated (RALLOC, Stata12) random intervention allocation will be provided for each participant.

#### Blinding

Given the nature of the intervention, it is not feasible to blind the participants to their group assignment. However, the P.A.R.T.Y. programs will be delivered independent of the research team, limiting the potential for the study to influence program content and conduct. Project personnel involved in randomisation will not participate in collection of outcomes data for the study cohort, conversely the personnel involved in collecting baseline and 12 month follow up data will not be involved in the randomisation of participants.

### Sample-size calculation

Similar studies have not previously been conducted in the ADF setting. Therefore, we based the effect-size on unpublished data provided on request from the RAN summarising the AUDIT scores and alcohol incident rates of RAN personnel. Published data show 1 in 5 (20%) ADF personnel report an AUDIT score of 8 or above, which represents hazardous or harmful alcohol consumption behaviour [15]. Our study was powered to detect an 8% lower prevalence of moderate to high risk behaviour (AUDIT score 8+) in the intervention groups relative to the control group with 80% power. To achieve this, 284 participants in each group is needed and this will be increased to 310 per group to allow for a 10% loss to follow-up.

### Study groups

#### In-hospital P.A.R.T.Y.

The In-Hospital P.A.R.T.Y. is a full day (6 h) harm minimisation and prevention program. Additional file 1: Appendix 2 describes the schedule of the in-hospital program. At the commencement of the program participants are provided with context talks from various health professionals, followed by interactive tours of the trauma centre, Intensive Care Unit and a ward area. These tours are facilitated by clinical staff in the areas and, in the case of the ICU and ward area, the participants also spend time with a family or patient. The afternoon is spent with Allied Health professionals who describe/show the effects of trauma to help participants better understand the consequences of disability following trauma. Overall, the program is designed to provide pertinent information to participants in order for them to be more aware of injury-producing situations, make informed prevention-orientated choices, and adopt behaviours and actions to minimise risk of injuries [7].

#### On-base P.A.R.T.Y.

The on-base P.A.R.T.Y. program is a four-hour adaptation of the in-hospital program. The content is consistent with the in-hospital program but the program is delivered by health professionals and trauma survivors, with the aid of simulation, in the health centre at the participating naval base. Additional file 1: Appendix 3 describes the schedule of the on-base program.

#### Annual awareness training

The ADF delivers a number of mandatory training modules to all personnel annually. One of these is an annual alcohol and other drug awareness program. The alcohol and other drug annual awareness training module is delivered by a designated and trained Alcohol and Other Drugs Program counsellor. This training typically consists of a PowerPoint presentation outlining the ADF/RAN responsibilities for all members and the organisation in relation to the sales and consumption of alcohol. The presentation takes approximately 1 h and is currently delivered as a face-to-face presentation.

### Measures

#### Primary outcome

The primary outcome measure will be the proportion of participants reporting an AUDIT score of 8 or above at 12 months’ post-intervention.

#### Secondary outcomes

The secondary outcome measure will be the number and time to alcohol related incidents in the 12 months’ post-intervention. All participants will be able to be followed up as a result of the ADF’s requirement of all members to report all civilian legal actions against them. This includes alcohol related offences.

#### Data analysis

Data will be analysed on a per-protocol and intention to treat basis. Summary statistics will be used to describe the difference between groups and ascertain if balance of groups has been achieved through the randomisation process. Frequencies and percentages will be used for categorical variables and mean and standard deviations, or median and inter-quartile range for continuous variables.

The primary outcome will be analysed using a modified Poisson regression model with a robust variable estimator [16]. The relative risk, and 95% confidence intervals (CI) of reporting an AUDIT score of 8 or above in the intervention groups will be calculated relative to the control group. Where necessary, the model will be adjusted for factors not balanced between the groups through the randomisation process.

The secondary outcome will be analysed using a Cox Proportional Hazards regression model assessing time to an alcohol related incident reported to the RAN, with participants not reported for an alcohol related incident censored at 12 months’ post-intervention. Where a participant leaves the RAN, these participants will be censored at the date of discharge from the RAN [16–18]. Hazard ratios, and 95% CI will be reported. For both analyses, multivariate models will be used if there is baseline imbalance between the groups.

## Discussion

The ADF forms part of a broader Australian community, so many factors that contribute to a potentially harmful drinking culture which exist in ADF are a reflection of those that occur in the broader Australian community [15]. To our knowledge this will be the first RCT investigating the efficacy of the P.A.R.T.Y. program in reducing risky drinking. The study will evaluate the effectiveness of two versions of the P.A.R.T.Y. program in young navy trainees. The findings of this project will allow evidence-informed decision-making related to further roll out of the program.

Our study is limited by our choice to include only participants who will only recently have joined the RAN, and exclude trainees who will have previously been identified as having had an alcohol related problem. We also acknowledge that whilst we will have recruited from the RAN’s largest training base, we are only considering trainees who will remain at this specific base for their ongoing training, missing trainees who will go on to complete their training on other Australian Defence Force bases.

## Conclusion

To our knowledge, our study will be the first RCT of the P.A.R.T.Y. since its inception in 1986. This is the first trial of the use of the P.A.R.T.Y. program in the military. Our study will determine the efficacy of two versions of the P.A.R.T.Y. program compared to usual training in a pragmatic RCT. Our design will allow us to make direct comparisons of the effects of the in-hospital program versus the on-base program. If the proposed intervention proves efficacious, it may be a useful program in the training of military personnel to reduce the prevalence of risky drinking behaviours and alcohol related incidents.

## Additional file

Additional file 1: Appendix 1. P.A.R.T.Y. Defence Attitudes and Behaviours Questionnaire. Appendix 2. P.A.R.T.Y. Defence In-Hospital Program. Appendix 3. P.A.R.T.Y. Defence On-Base program. (DOCX 1173 kb)

## Abbreviations

ADF: Australian Defence Force
AODP: Alcohol and Other Drug Program
AUDIT: Alcohol Use Disorders Identification Test
HMAS: Her Majesty’s Australian Ship
IET: Initial Entry Training
MDMQ-R: Modified Drinking Motives Questionnaire – Revised
P.A.R.T.Y. Defence A&BQ: Prevent Alcohol and Risk-related Trauma in Youth Defence Attitudes and Behaviours Questionnaire
P.A.R.T.Y.: Prevent Alcohol and Risk-related Trauma in Youth
RAN: Royal Australian Navy.
