# Supplementary material for: Measuring the effectiveness of in-hospital and on-base Prevent Alcohol and Risk-related Trauma in Youth (P.A.R.T.Y.) programs on reducing alcohol related harms in naval trainees: P.A.R.T.Y. Defence study protocol
Source: BMC Public Health. 2017 May 2;17:380. doi: 10.1186/s12889-017-4330-8 (PMC5414328; doi:10.1186/s12889-017-4330-8)
Supplement: Additional file 1: — Appendix 1. P.A.R.T.Y. Defence Attitudes and Behaviours Questionnaire. Appendix 2. P.A.R.T.Y. Defence In-Hospital Program. Appendix 3. P.A.R.T.Y. Defence On-Base program. (DOCX 1173 kb) [file 12889_2017_4330_MOESM1_ESM.docx]

Appendix 1: P.A.R.T.Y. Defence Attitudes and Behaviours Questionnaire

Appendix 2: P.A.R.T.Y. Defence In-Hospital Program

Appendix 3: P.A.R.T.Y. Defence On-Base program

##
